# Camphor-mediated synthesis of carbon nanoparticles, graphitic shell encapsulated carbon nanocubes and carbon dots for bioimaging

**DOI:** 10.1038/srep21286

**Published:** 2016-02-24

**Authors:** Goldie Oza, M. Ravichandran, Victor-Ishrayelu Merupo, Sachin Shinde, Ashmi Mewada, Jose Tapia Ramirez, S. Velumani, Madhuri Sharon, Maheshwar Sharon

**Affiliations:** 1 Department of Electrical Engineering, CINVESTAV-IPN, Mexico D.F; 2 Program on Nanoscience and Nanotechnology, CINVESTAV-IPN, Mexico D.F; 3 Department of Genetics and Molecular Biology, CINVESTAV-IPN, Mexico D.F; 4 Nagoya Institute of Technology, Nagoya, Japan; 5 Walchand Centre for Research in Nanotechnology and Bionanotechnology (wcRnb), Walchand College of Arts and Science, Walchand-Hirachand Marg, Ashok Chowk, Solapur-413006 MS, India; 6 Institute of Molecules & Materials of Le Mans (IMMM) UMR CNRS 6283, Universite du Maine, 72085 Le Mans, France

## Abstract

A green method for an efficient synthesis of water-soluble carbon nanoparticles (CNPs), graphitic shell encapsulated carbon nanocubes (CNCs), Carbon dots (CDs) using Camphor (*Cinnamomum camphora)* is demonstrated. Here, we describe a competent molecular fusion and fission route for step-wise synthesis of CDs. Camphor on acidification and carbonization forms CNPs, which on alkaline hydrolysis form CNCs that are encapsulated by thick graphitic layers and on further reduction by sodium borohydride yielded CDs. Though excitation wavelength dependent photoluminescence is observed in all the three carbon nanostructures, CDs possess enhanced photoluminescent properties due to more defective carbonaceous structures. The surface hydroxyl and carboxyl functional groups make them water soluble in nature. They possess excellent photostability, higher quantum yield, increased absorption, decreased cytotoxicity and hence can be utilized as a proficient bio imaging agent.

The remarkable features of photoluminescent nanocarbon family are attributed to their water solubility, good quantum yield, low photobleaching, high biocompatibility and efficient biolabelling agent^1^. They are far more superior to other conventional fluorescent organic dyes and luminescent inorganic cadmium based-quantum dots in terms of photostability and toxicity. Carbon dots (CDs) are one of carbon based nanoallotrope, which has now received major attention since it possesses unique optical, optoelectronic properties exhibiting size and excitation wavelength dependent photoluminescence^1^. These properties are purely due to the most well-known fact that they exhibit quantum confinement effect, emissive traps and edge effects which exists on their surface. They have been synthesized using both top-down and bottom-up methods. The methods used for their synthesis are laser ablation^2^, arc discharge^3^, electrochemical oxidation^4^, microwave^5^, hydrothermal treatment^6^, ultrasonic method^7^ and other supported methods^8^. They are considered to be insulating and disordered nanostructures under the family of highly conducting crystalline graphene. The latter is unique due to its electronic structure with linear dispersion of Dirac electrons. The defective characteristics of CDs doesn’t allow material scientists to observe its fundamental 2-dimensional condensed-matter effects, but has gathered attention because of its heterogeneous chemical and electronic structures that can be altered in the solution due to the availability of oxygen-containing functional groups at the edge. There can be drastic transformation of CDs from an insulator to semi-conducting metal by tuning the ratio of sp^2^ and sp^3^ fractions leading to the generation of bandgap. Graphene has zero-band gap, but CDs are electronically active material containing both π-states from sp^2^ carbon and a large carrier transport gap between the σ-states of its sp^3^-bonded carbon^8^. It becomes very easy to get size controlled synthesis of CDs if the starting raw material already possesses small domain structure of the sp^2^ carbons and these nanostructures can be isolated efficiently.

In the present work, camphor is efficiently exploited for the formation of CD synthesis, which allows both hexagonal and pentagonal-ringed structure without the need of getting completely oxidized to individual carbon atoms. Camphor imparts both these reactive rings as compared to the conventional precursors which possess only planar hexagonal rings as templates for nanoparticle formation. Hence, camphor is more preferred over other graphitic materials for synthesizing different sizes and shapes of carbon nanostructures^9^.

Camphor is a latex of C*innamomum camphora* that has been exploited for decades, as a precursor for synthesis of fullerenes^10^, carbon tubules^11^, glassy carbon^12^, diamond-like carbon^13^, carbon nanobeads^14^ semiconducting carbon^15^. and single/multi-walled carbon nanotubes^16^. Monolayer^17^, few layered and multi-layered graphene sheets^18^ are all synthesized from camphor which are exploited for plethora of applications such as designing a transparent electrode^19^, photovoltaic cell^20^ etc. All the above substances are chemically inert, less toxic but do not possess very high luminescent properties. Thus, there is a need to synthesize carbon dots, so that they can be exploited in imaging, sensors and drug-delivery.

In this report, we demonstrate a facile synthesis of CDs from camphor which is known to be an insect repellent and disinfectant in sweets. To the best of our knowledge, this is the first report in which a natural extract, camphor has been exploited to synthesize water soluble CDs using 3 steps: acid oxidation method with an aid of nitric and sulphuric acids, alkaline hydrolysis by sodium hydroxide and sodium borohydride reduction leading to the formation of CDs and further enhancement of its photoluminescence. The unique signature marker of successful synthesis of CDs is its blue photoluminescence under UV light and size, which is less than 10 nm. The photoluminescence (PL) modus operandi is confirmed by the quantum confinement effect or conjugated π-domains determined by carbon core and different emissive traps existing on the surface of CDs. The step by step reaction involves CNP formation on acid oxidation of camphor and on further treatment with sodium hydroxide causes enhancement of blue photoluminescence to form CNCs. This was then reduced by sodium borohydride to give bluish green-fluorescent CDs. All the three carbon nanostructures are highly photostable, resistant to photobleaching, monodisperse, water soluble, cytocompatible, stable in physiological saline, non-toxic, exhibit high photoluminescence thus proving to be efficient bioimaging agents.

## Results

Camphor (C_10_H_16_O) is a molecule which possess three CH_3_ groups and one C=O group (the elemental compositional is C-76.28% & H-8.6% (Table S1)). It is reported that after acid oxidation, partial combustion or pyrolysis, three methyl groups are cleaved, leading to the formation of reactive skeleton of camphor molecules comprising of one pentagonal and a hexagonal ring^9^. These highly reactive rings are considered to be basic templates for spherical shaped CNPs, CNCs and CDs.

### Structural and morphological properties

**Figure 1:**
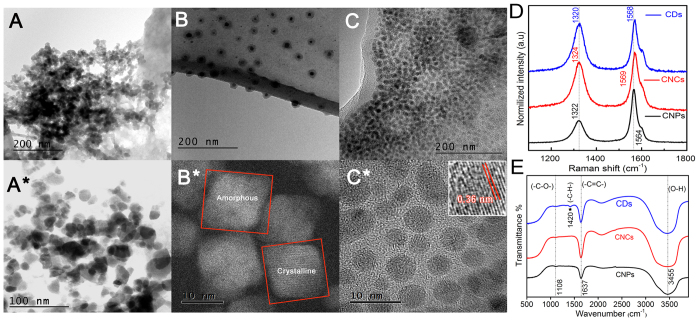
TEM, HR-TEM, HAADF-STEM, Raman and FTIR of CNPs, CNCs and CDs. (**A**) TEM image of carbon nanoparticles (CNPs) interconnected to form a network, (A*) HR-TEM image of CNPs, (**B**) TEM image of Carbon nanocubes (CNCs) encapsulated by graphitic shell, the spherical nuclei are encircled by red color spheres which acts as a template for carbon nanostructure formation and further graphitic shell coated carbon nanocubes are in a square-shaped red coloured brackets, (B*) HAADF-STEM images of intermediate step of nanocuboidal seeds on which graphitic shells are formed, both amorphous and crystalline nanostructures are marked with red coloured square brackets (**C**) HR-TEM image of CDs, (C*) HAADF-STEM image of CDs, after water-ethanol-chloroform centrifugation and dialysis, (inset shows IFFT of carbon dots exhibiting a lattice fringe width of 0.36 nm. (**D**) Raman spectra of CNPs, CNCs and CDs, which shows D as well as G band, (**E**) FTIR spectra of CNPs, CNCs and CDs exhibiting -C-H bending, -C=C- stretching and -O-H stretching, -C-O stretching.

CNP formation from acid oxidation method of camphor was demonstrated from the TEM images, exhibited in Fig. 1A. The cross linking between CNPs has led to the formation of a network glued by multiple weakly bonded Van der Waals force. Such fettered or agglomerated carbon nanoparticles of size 15–20 nm, can form beaded chain like structures. There are unreacted agglomerates as seen in Fig. 1A which is a clear indication of incomplete or partial oxidation of camphor precursor. The surface energies of CNPs are so high that it leads to the formation of large conglomerates tethered to each other through dispersive forces. Oxygen atoms in camphor molecules due to intense oxidative process lead to the formation of puckered ring structures, thus forming a scaffold for CNPs through molecular fusion route.The growth of carbon particles once initiated, gets terminated at a single point on the surface of another particle, present in its juxtaposed position. Such interconnected viaducts of CNPs were observed to be overlapped by each other. These particles after dialysis showed fewer amounts of partially oxidized camphoric intermediates, which is also confirmed by HR-TEM images (Fig. 1A*).

TEM micrograph shown in Fig. 1B presents two types of carbon nanostructures after alkaline hydrolysis of CNPs with an aim to synthesize CDs:

- (1) Very small carbon dots of size 2–6 nm (marked by red coloured circles).
- (2) CNCs of size 10–15 nm and a concentric layer of graphitic shell of 2–5 nm on its surface (marked by red coloured square brackets).

**Figure 2:**
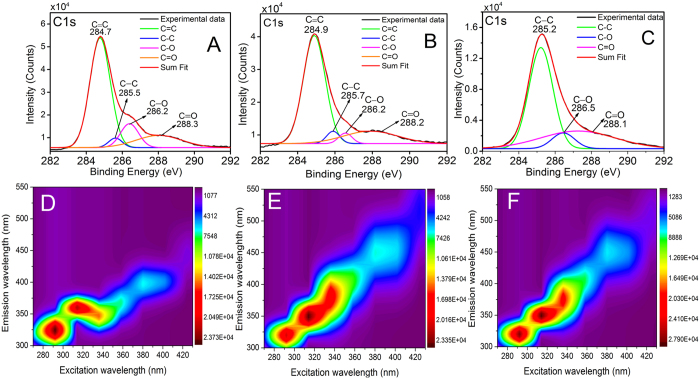
XPS analysis, excitation-emission Photoluminescence contour plots of CNPs, CNCs and CDs. (**A**–**C**) shows the high-resolution spectra of C1s in CNPs, CNCs and CDs respectively, exhibiting 3 peaks in the range of 284–288 eV binding energies exhibiting C=C, C-C, C-O/C=O, (**D**–**F**) are the contour plots of the excitation-emission PL matrix for CNPs, CNCs and CDs, respectively.

It is also observed that two CNCs encapsulated by graphitic concentric shell have fused together their respective carbonaceous graphitic layers. This is in accordance with Sharon *et al*. who have explained the complete mechanism of such graphitic shell formation^14^. It is found that CNCs are monodisperse without any agglomeration, whereas previously Sharon *et al*. have shown spongy carbon nanobeads of very high aggregations. According to TEM, it is clearly seen that acid oxidized CNPs on hydrolysis with NaOH at 100 ^°^C undergoes recrystallization via molecular fission and fusion routes. In this method, the process is reinitiated by further disaggregation of CNPs into minimum size of 2–5 nm which is seen in Fig. 1B. These small particles after a point starts acting like nuclei, (encircled with red color in Fig. 1B) and the transformation phase can be seen between CNPs to CNCs (supplementary Fig. S1A,B), which then starts growing to form cuboidal structures of size 10–15 nm. This can also be confirmed from high angle annular dark field-Scanning transmission electron microscopy (HAADF-STEM) image in Fig. 1B*. It is observed that there are two kinds of CNCs, one is amorphous and the other crystalline. Thicker and denser graphitic shell then starts growing on such a nanocube. The mechanism for the nucleation of carbon nanostructures, deposition of carbon on the nuclei, and finally formation or growth of graphene shells can be explained by the template growth model. In the previous reports based on molecular dynamic simulations, it was shown that, for obtaining curvature equivalent to small nanoparticles of size less than 2 nm, the folding of graphite sheets (which depends on the size and curvature of graphite sheets) need high activation energy of around 1–9 eV. However, considering the long-range interaction of carbon atoms in graphite sheets as well as carbon atoms within the particle, it is evident that the template growth of surface graphene layers is a thermodynamically favourable process, particularly when the radius of the template is greater than 8 nm^21^. The diffusion and dissolution of carbon atoms in carbon nuclei is a favourable modus operandi and hence the template growth mechanism has won over most of the other models since the initial nuclei can act as a template for the growth of particles achieving cuboidal shapes. It has been already proved that C60/70 when present in the solution is very critical for the synthesis of graphitized structures of graphene. It can also be noted that the graphitized structures are efficient building blocks for a large-scale synthesis of graphene structures. Hence, as soon as CNPs are synthesized in the first step, during hydrolysis, the web of particles starts disintegrating and acts as nuclei for the growth of particles as a whole and then graphitic shell encapsulate them. This can be corroborated from a generalized mechanism in which thermal treatments or strong electron irradiations can curl up the planar sheets to form graphitic shells for the elimination of dangling bonds present in the planar graphitic sheets^22^. The shell formation is possible under favorable sp^2^ carbon network, which is present in carbon nanoparticles as confirmed from the XPS analysis (Fig. 2A). Finally to enhance fluorescence, we further reduced CNCs using sodium borohydride at 100 ^°^C. In the intermediate stage, it is found that there are few particles covered by cuboidal graphitic shell (supplementary Fig. S1B,C). But after 1 hr of heating and 2 hrs of mild ultrasonication (500 W, 40 kHz), this mixture was dialyzed using a 3.5 kDa bag for 24 hrs to form uniformly distributed spherical particles of size 2–8 nm (Fig. 1C). Figure 1C* shows high-resolution transmission electron microscope image (HR-TEM) of dialysed CDs, revealing high crystallinity. The lattice parameter of 0.36 nm as shown in the IFFT (inset) is the d-spacing between the layers, considering that the bulk graphite has d-spacing of 0.36 nm.

X-ray diffraction (XRD) patterns of the CNPs, CNCs and CDs are displayed in supplementary Fig. S2. Both CNPs and CNCs showed a broad peak in the range of 21–23^°^ that corresponds to the graphitic structures^23^. The dialyzed sample of CDs possesses a peak at 25.23^°^ which also matches with bulk graphite. This small difference in d spacing between the bulk graphite and CDs is attributed to the turbostratic stacking of carbon structures and the presence of − OH, − CH at the edges that are responsible for the enlargement of the spacing of graphene layers^23^.

Elemental analysis (Supplementary Table S1) was performed to understand the carbon content of all the three kinds of structures as compared to the precursor. The analysis showed that CNP comprises of 80.76 wt% carbon, 3.72 wt% hydrogen as compared to camphor which is 78.90 wt% carbon and 10.59 wt% hydrogen. The increase in the carbon content shows that there is carbonization after acid oxidation method. CNCs showed 83.36 wt% of carbon and 5.49 wt% of hydrogen, which confirms the reduction of CNPs. Furthermore, on sodium borohydride reduction, CDs showed 86.12 wt% carbon and 7.76 wt% hydrogen. The increase in hydrogen content clearly shows that the carbonyl groups were reduced to hydroxyl groups by NaBH_4_^24^.

### Photophysical and chemical properties

Raman spectroscopy is a non-destructive method to characterize crystalline, nanocrystalline and amorphous carbons^25^. Raman spectrum of pure graphite sample shows single G peak around 1550–1600 cm^−1^. But as the disorderness and defects rises in the pure graphitic sample, there is an evolution of a D band in the range of 1300–1350 cm^−1^ which is also known as breathing mode. This D band possess both first order and second order resonance processes and exists because of the breathing modes of six atom rings^26^,^27^. CNPs, CNCs and CDs possess G band at 1564, 1569 and 1568 cm^−1^ while D band at 1322, 1324 and 1320 cm^−1^ respectively (Fig. 1D). This G peak corresponds to E_2g_ phonon at the Brillouin zone and is due to the sp^2^ clusters. The D peak corresponds to the A_1g_ phonon breathing mode and is activated via defects arising from the transverse optical (TO) phonons near the K points exactly at the first Brillouin zone^28^. D band is attributed to disordered sp^3^ carbon. This band is quite dispersive with an aid of excitation energy which occurs due to the Kohn Anomaly at K^29^. The existence of D′ peak centered at ~1605 cm^−1^ is due to the intervalley double resonance process around K or K′^30^. The Raman spectrum of these carbon nanostructures is quite similar to graphene oxide sheets, which clearly demonstrates that they are comprised of small sp^2^ clusters surrounded by a disordered sp^3^ carbon matrix^31^,^32^. This is manifested by Tuinstra-Koenig relationship (Supplementary equation 1a & b), which correlates the intensity ratios of D and G peaks (I_D_/I_G_) with the in-plane correlation length^26^. I_D_/I_G_ increases as sp^2^ cluster size decreases, while D-mode becomes dominantly active. In Fig. 1D, the ratios of I_D_/I_G_ for CNPs, CNCs and CDs are calculated as 0.436, 0.8315 and 0.936 respectively. It is observed that increased I_D_/I_G_ ratio correlates with sp^3^/sp^2^ carbon and is a clear indication of structural defect^33^. As per T-K relation, the sp^2^ cluster size of CDs is 3.74 nm (I_D_/I_G_-0.936), while the average size of the particle from HR-TEM is 8 nm. The crystal size from HR-TEM is greater than the sp^2^ clusters, thus confirming that they are made up of small sp^2^ clusters inside a disordered sp^3^ carbon matrix. This shows that after the sodium borohydride reduction, there is formation of more defective carbonaceous structures. The fluorescence is attributed to surface defects which entrap excited state energy, thus leading to enhanced PL. Further, the mechanism of photoluminescence is explained later in another section (Photoluminescence mechanism), comprehending the deeper intricacies of borohydride reduction leading to enhanced PL.

CNPs, CNCs and CDs possess -C=C- symmetrical stretching vibration at 1637 cm^−1^ as displayed by FTIR in Fig. 1E thus indicating that the core of the particles are made up of a combination of sp^3^ and sp^2^ hybridized carbon as is also confirmed from Raman spectra. As mentioned earlier, camphor is a terpenoid having three methyl groups and one carbonyl group. It gets degraded on oxidation and undergoes carbonization, thus forming first the nucleus of both alkene and alkane chain combinations. Then as heating time increases, the surface edges start getting a passivation layer of -O-H (hydroxyl) and -C-O (carbonyl) groups as indicated by 3455 cm^−1^ and 1108 cm^−1^ respectively, which may be responsible for making them water soluble as compared to camphor that is poorly soluble in water. This self-passivated layer on the surface of carbon nanostructures render photoluminescent properties. CDs also possess a weak bending vibration of CH_2_ absorption at 1420 cm^−1^, thus confirming the reduction by sodium borohydride. The hydroxyl group is also broadened in CDs as compared to CNPs and CNCs indicating more surface passivation of hydroxyl groups in the reduced nanostructures^34^. FTIR was performed at different dilution ratios ranging from 1:5 to 1:100 for all the three carbon structures, but we could not find much difference in the spectra of undiluted samples and the diluted ones (supplementary Fig. S3).

**Figure 3:**
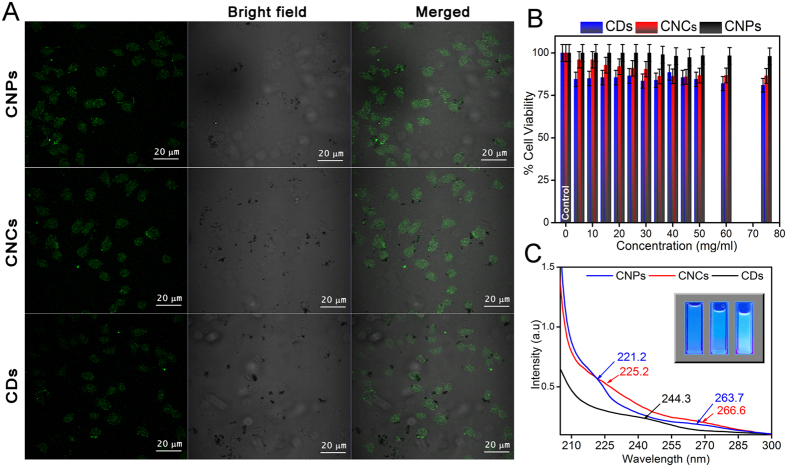
Confocal microscopy images, MTT assay of SHSY5Y cells incubated with CNPs, CNCs and CDs and UV-Visible spectra. (**A**) Fluorescent images of CNPs, CNCs and CDs are collected at the excitation wavelength of 405 nm, both bright field and merged images are shown. (**B**) MTT assay of CNPs, CNCs and CDs, showing cell viability with error bars at different concentrations ranging from 5 to 75 mg/ml for 24 hrs, (**C**) UV-Visible spectra with fluorescent images (Inset) of CNPs, CNCs and CDs are shown.

Further, functional groups on the surface of CNPs, CNCs and CDs were studied using X-ray photoelectron spectroscopy (XPS) to assess the surface composition and binding energies. Figure 2A–C depicts deconvoluted C1s peak of all the 3 samples. C1s peaks are dominantly present, thus confirming the presence of carbon in all the samples^24^. The different peaks present in C1s refers to the C=C bond (284.7–284.9 eV), which is attributed to sp^2^ hybridized carbon, while C−C (285.2–285.5 eV), C−O (286.2–286.5 eV) and C=O (288.1–288.3 eV) bonds are attributed to sp^3^ hybridized carbons. This is in accordance with FTIR and Raman analysis. XPS confirms that all the three kinds of carbon particles are passivated by hydroxyl and carboxyl groups. These functional groups improve the solubility and stability of all the three kinds of carbon nanostructures in an aqueous system. UV-Visible spectroscopic measurement shows a characteristic broad peak at 221.2 nm and an absorption shoulder at 263.7 nm for CNPs. Similarly, CNCs show peaks at 225.2 and 266.6 nm. The peaks at 221.2 nm (CNPs) and 225.2 nm (CNCs) are due to the π-π* transition of the C=C band and the peaks at 263.7 nm (CNPs) and 266.6 nm (CNCs) are due to n-π* transition of the conjugated C=O (Fig. 3C)^23^. Camphor on acidic oxidation undergoes graphitization and a peak at 221.2 nm is attributed to the aromatic sp^2^ domains^35^. Due to nitric acid and sulphuric acid treatment, CNPs are passivated by carbonyl and carboxyl groups which are attributed by the absorption band at 263.7 nm. This is also observed in CNCs and is confirmed by the absorption at 266.6 nm. Both the absorption bands corresponding to the sp^2^ core as well as surface passivation is also confirmed by FTIR and XPS as discussed in previous section. CDs exhibit an absorption at 244.3 nm which shows a red shift from ~220 nm as compared to CNPs and CNCs. It is speculated that this new absorption peak is due to structural alterations in the sodium borohydride reduced CDs. The absorption shoulder around ~260 nm disappears on borohydride reduction, which may be due to the decrement in the carboxyl groups on the surface^36^,^37^.

The unique photoluminescence behaviour of all the three structures was also explored. The explanation of PL behaviour is as follows:

- (1) CNPs show weak blue luminescence after the treatment of nitric acid and sulphuric acid. This blue luminescence is due to strong quantum confinement and edge effects, which is observed even at 100 nm, but becomes more dominant when the sizes are cracked down further^38^. The PL spectrum of CNPs exhibit excitation-dependent mechanism as displayed in the excitation-emission-contour plot (Fig. 2D). The most noticeable excitation/emission plot is derived at 290/324 nm. When the excitation wavelength is increased from 290 to 450 nm, the PL peak shifts towards longer wavelengths and there is a rapid decrement in the intensity of the peak (Fig. 2D). The 2-D PL spectrum (Supplementary Fig. S4A) shows a strong peak at 324.5 nm (3.82 eV) when excited by an absorption band of 290 nm (4.26 eV), with a Stokes shift of 34.5 nm (0.44 eV). The PL excitation (PLE) spectrum recorded with the strongest emission, exhibited a sharp peak at 290 nm. The surface passivation of hydroxyl and carbonyl groups play a significant role in exhibiting PL emission, but as CNPs possess less number of such functional groups, they are not completely passivated^39^ and exhibits a quantum yield of 8.6% (Supplementary equation 2).
- (2) CNCs formed by sodium hydroxide treatment of CNPs shows strong blue luminescence under both alkaline and neutral condition. CNCs also show excitation dependent mechanism, as excitation wavelength has profound effect on PL peak shift (Fig. 2E). The most noteworthy excitation/emission contour plot is produced at 310/348.4 nm. The 2-D plot (supplementary Fig. S4B) exhibits a strong PL peak at 348.4 nm (3.55 eV) when excited by an absorption peak at 310 nm (3.99 eV), thus displaying a Stokes shift of 38.4 nm (0.44 eV). CNCs demonstrated a strong PLE peak at 310 nm and its quantum yield was measured to be 15.60%.
- (3) CDs are formed when CNCs are reduced by sodium borohydride, showing strong bluish-green fluorescence under neutral condition. CDs also show excitation dependent mechanism, which is exhibited from the excitation-emission contour plot (Fig. 2F). The excitation-emission maxima is obtained at 310/352.5 nm. The 2-D plot (Supplementary Fig. S4C) also displays a strong PL peak at 352 nm (3.51 eV), when excited by an absorption peak at 310 nm (3.99 eV), having a Stokes shift of 42 nm (0.48 eV). The quantum yield of CDs was tremendously increased to 35.26%. This manifests that sodium borohydride reduction regulates the concentration of isolated sp^2^ clusters, thus increasing the PL intensity by manifold as compared to CNPs and CNCs^37^.

The higher quantum yield observed in CDs as compared to CNCs and CNPs can also be correlated with the size of the sp^2^ fragment. It has been reported that HOMO-LUMO gap is dependent on the size of the sp^2^ domain. There is gradual decrement in the gap, as the fragment size increases^40^. In the present study, it is shown that the sp^2^ cluster size decreases from 8.036 nm for CNPs to 3.47 nm for CDs. Furthermore, the gap increases from 0.44 nm to 0.48 nm. Hence, it can be concluded that the strong emission exhibited by CDs is predominantly due to the quantum-sized graphitic structure.

We also noted that CNPs, CNCs and CDs are sensitive to pH ranging from acidic to alkaline region (pH~1 to ~12). When pH is changed from 12 to 1, the absorption of CNPs, CNCs and CDs all show minor red-shift as shown in Supplementary Fig. S5. Moreover, PL emission is maximum at pH ~12, while it is quenched at pH 1. This shows that they possess Lewis basic sites which is quenched in the acidic media and is restored in the basic media.

The main application of CNPs, CNCs and CDs in bioimaging could be fulfilled if they show stability in different physiological pH such as neutral condition, weakly acidic and weakly basic. It was found that they exhibited similar PL intensities at all pH ranging from 3 to 9, thus confirming their tolerance and stability towards weakly acidic and weakly basic pH as depicted in Supplementary Fig. S6A, which shows fluorescence emission spectra (λ_ex_−290 nm) at different pH ranging from 3 to 9 for CNPs. Figure S6B,C corresponds to fluorescence emission spectra (λ_ex_−310 nm) at different pH ranging from 3 to 9 for CNCs and CDs respectively. Likewise all the three types of carbon nanostructures exhibited good photostability for more than 1000 s at an excitation wavelength of 290 nm for CNPs in Supplementary Fig. S6D. Photoemission stability of CNCs and CDs at λ_ex_−310 nm for 1000 s, is also given in Supplementary Fig. S6D thus confirming their efficiencies to be exploited in Bioimaging^41^.

### Cellular Imaging

Biocompatibility is an important criterion for a bioimaging agent. The cytotoxicity of CNPs, CNCs and CDs is evaluated on SHSY5Y cells at different concentrations ranging from 5 to 75 mg/ml for 24 hrs. (Fig. 3B). The cellular toxicity of the carbon nanostructures are dependent upon size and shape of the particles. Moreover, agglomeration also has a great impact on the distribution and uptake of the nanoparticles. It was found that all the three structures were non-toxic to cells, irrespective of their size and shape. The mechanism for cellular uptake of carbon nanoparticles and carbon dots are still unknown, but a general modus operandi for *in vitro* cellular uptake of gold nanoparticles of dimensions less than 100 nm is well- studied and is found to be receptor-mediated endocytosis (RME)^43^. As per the “wrapping effect model”^44^ for cellular uptake of nanoparticles, two factors are responsible for how fast and how many number of particles can enter inside the cellular milieu:

- (1) Free energy or thermodynamics that leads to receptor ligand interaction,
- (2) Receptor-ligand diffusion kinetics after the wrapping of the nanoparticles.

Hence, it is considered that CNPs, CNCs and CDs enter inside the cells via RME and that the particles are distributed based on the “wrapping effect model”.

Due to non-toxic behavior, excellent fluorescent property, high photo stability, water solubility of CNPs, CNCs and CDs having multitudinous functional groups, they are considered to be an attractive alternative to fluorescent dye for bioimaging. All the three types of particles were studied under confocal microscopy with SHSY5Y (neuroblastoma) cells for their comprehension of the fluorescent properties in the absence of any fluorescent dyes. The cells were incubated with the three kinds of nanostructures for 4 hrs at 37 °C. Under confocal microscopy, the cells do not show any nuclear or cytoplasmic morphological change (Fig. 3A). In addition, they exhibited fluorescence (Fig. 3A) in both cytoplasmic regions and on cellular membrane^45^. It is clear that all the three particles are properly dispersed in nucleus and cytoplasm. Thus, it is inferred that all the three structures reported can be employed as an excellent fluorescent marker for biomedical applications. In case of control cells, we could not visualize the green fluorescence under microscope.

### Formation Mechanism of carbon nanostructures

**Figure 4:**
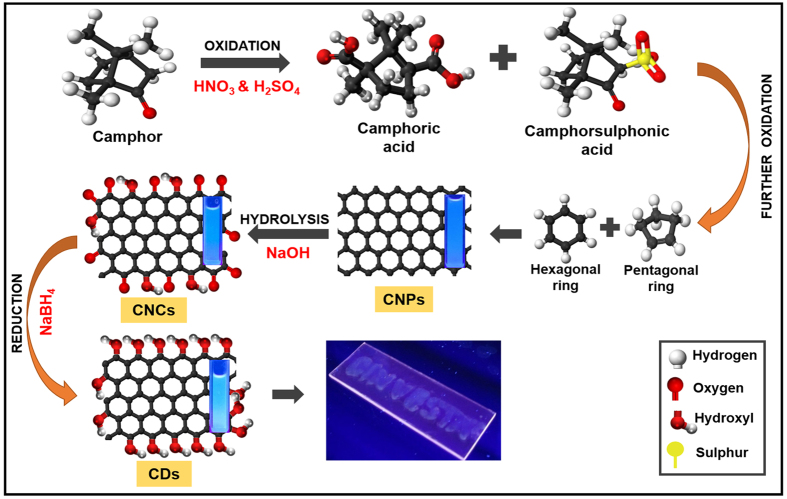
Schematic representation for step-wise transformation of camphor to carbon dot.

The mechanistic determination for CD formation is another challenging task, which is achieved using Gas chromatography-Mass Spectrometry (GC-MS) and ^1^H-NMR. In the schematic representation (Fig. 4), it is demonstrated that camphor on acid oxidation by nitric acid and sulphuric acid forms orange coloured liquid comprising of camphoric acid and camphor sulfonic acid. The presence of camphor, camphoric acid and camphor sulfonic acid was confirmed by GC-MS (supplementary Fig. S7). It is speculated that on further oxidation, such intermediates dissociate to form hexagonal and pentagonal radicals. These two radicals form the scaffold for the formation of CNPs which is also catered by high temperature and harsh oxidative acidic conditions^9^. One of the reaction intermediates formed is *1b,5,5,6a-Tetramethyl-octahydro-1-oxa-cyclopropa[a]inden-6-one* as shown in supplementary Fig. S8 CNPs. Such CNPs get hydrophilicity due to the introduction of hydroxyl and carboxyl groups on the surface caused by the acidic treatment. This leads to their stability when dispersed in polar solvents such as water and ethanol^46^. CNPs undergo hydrolysis on treatment with sodium hydroxide to form more hydroxyl edges on the surface and such structures were named as CNCs. This solution has one of the reaction intermediates as acetaldehyde, *3,3-dimethylcyclohexylidene)-(E)* as shown in supplementary Fig. S8 CNCs. To enhance the quantum yield, the hydrolyzed solution was further reduced using sodium borohydride. Likewise, hydroxyl groups are abundantly formed on the surface as indicated by GC-MS from the formation of intermediate *cyclohexanol, 2-methyl-5-(1-methylethenyl)* in supplementary Fig. S8 CDs. The ^1^H-NMR (Supplementary Fig. S9) spectra depicts three different types of chemical environments of camphor, CNPs and CNCs. The presence of sp^2^ and sp^3^ carbons in both CNPs and CNCs is further confirmed from ^1^H-NMR, which shows three regions:1–3 ppm (for sp^3^ C-H protons), 3–4 ppm (for the protons attached with hydroxyl, ether and carbonyl groups), 6–8 ppm (for the aromatic or sp^2^ protons)^47^.

### Mechanism of Photoluminescence

The photoluminescence mechanism is evident from Raman spectrum and NMR, that a major fraction of carbon in CNPs, CNCs and CDs are sp^3^ hybridized and are covalently bonded with epoxy and hydroxyl groups. The residual carbon is sp^2^ hybridized, which is either bonded with the neighbouring carbon atom or with oxygen, thus forming carboxyl or carbonyl groups. Such decorations are predominantly seen on the surface edges of such carbon nanostructures. But as CNPs are transformed into CDs, the sp^3^/sp^2^ fractions increase, thus unlocking many possibilities for new functionalities. It is determined that such carbon nanostructures exhibit PL due to geminate recombination of localized electron-hole pairs in sp^2^ clusters. These recombination centers behave like a chromophoric luminescent region, thus tunable PL emission is possible by the regulation of such sp^2^ sites. Hence, PL is purely dependent on the sp^2^ fraction in disordered carbon system^48^,^49^. Here, in this article, it is manifested that by regulating the sp^2^ cluster concentration, through sodium borohydride reduction, PL intensity is enhanced.

All these three types of carbon viz., CNPs, CNCs and CDs possess isolated sp^2^ clusters inside the carbon-oxygen sp^3^ matrix causing localization of electron-hole pairs, thus leading to radiative recombination of small clusters.

To summarize, we have demonstrated that a natural precursor, camphor can be utilized to synthesize carbon nanoparticles (CNPs) using a simple acid oxidation method, which on alkaline hydrolysis forms carbon nanocubes (CNCs) and furthermore on reduction of CNCs forms carbon dots (CDs). All the three particles display a broad absorption spectrum from UV to visible region, good water solubility, good cytocompatibility, high pH and photo-stability, high quantum yield, proficient bioimaging agent.

## Additional Information

**How to cite this article**: Oza, G. *et al.* Camphor-mediated synthesis of carbon nanoparticles, graphitic shell encapsulated carbon nanocubes and carbon dots for bioimaging. *Sci. Rep.* **6**, 21286; doi: 10.1038/srep21286 (2016).

## Supplementary Material

Supplementary Information
