# Supplementary material for: Camphor-mediated synthesis of carbon nanoparticles, graphitic shell encapsulated carbon nanocubes and carbon dots for bioimaging
Source: Sci Rep. 2016 Feb 24;6:21286. doi: 10.1038/srep21286 (PMC4764906; doi:10.1038/srep21286)
Supplement: Supplementary Information [file srep21286-s1.doc]

**Camphor-mediated synthesis of carbon nanoparticles, graphitic shell encapsulated carbon nanocubes and carbon dots for bioimaging**

Goldie Oza^a§^, M. Ravichandran^b^, Victor-Ishrayelu Merupo^a^, Sachin Shinde^d^, Ashmi Mewada^e^, Jose Tapia Ramirez^c§^, S. Velumani^a,b§^, Madhuri Sharon^e§^, Maheshwar Sharon^e^

^a^Department of Electrical Engineering, ^b^Program on Nanoscience and Nanotechnology, ^c^Department of Genetics and Molecular Biology, CINVESTAV-IPN, Mexico D.F, ^d^Nagoya Institute of Technology, Nagoya, Japan, ^e^Walchand Centre for Research in Nanotechnology and Bionanotechnology (wcRnb), Walchand College of Arts and Science, Walchand-Hirachand Marg, Ashok Chowk, Solapur-413006 MS, India

^§^Correspondence and requests for materials should be addressed to:

S.V. (velu@cinvestav.mx), J. T. R. (jtapia@cinvestav.mx), M.S. (sharonmadhuri@gmail.com) and G.O. (goldieoza@gmail.com)

**Supplementary Information**

**Materials:**

Analytical-grade Camphor, Sulphuric acid, Nitric acid, Tryptophan, Sodium hydroxide, Sodium borohydride, penicillin (100 μg/mL), streptomycin (100 μg/mL), 3-(4,5-Dimethylthiazol-2-yl)-2,5-diphenyltetrazolium bromide (MTT) and Non-essential amino acid solution were purchased from Sigma Aldrich and used as received. Eagle's Minimum Essential Medium (EMEM), F12 Medium (nutrient mixture) and fetal bovine serum (FBS) were procured from Gibco Company. High-purity water with the resistivity of greater than 18 M.cm^-1^ was used in the experiments. Dialysis bags of MW (3.5 KDa) were used to separate small sized fraction.

**Synthesis & purification of Carbon dots (CDs):**

5 gms of Camphor was dissolved in 15 ml of Sulphuric acid and 5 ml of Nitric acid (H_2_SO_4_: HNO_3_ - 3:1), which turned into an orange coloured liquid. This solution was heated at 100°C till the whole solution turned into a black carbonized material, which was dissolved in 10 ml deionized water. Such solution was labelled as carbon nanoparticles (CNPs). They were further treated by 5 M sodium hydroxide at 100°C and were labelled as CNCs. CNCs were then treated by 10 mM Sodium borohydride at 100°C and then ultra-sonicated for 2 hrs (500 W, 40 kHz). This mixture was dialyzed using a 3.5 kDa bag for 24 hrs, and labelled as CDs. Both CNPs and CNCs were centrifuged at 5000 rpm to remove larger particles, and then the supernatant was used for further analysis. All the above experiments were performed in a benchtop acid digestion fume hood.

**Characterization & Instruments:**

Low resolution TEM images were examined with JEOL JEM-2100 transmission electron microscope (TEM). And high resolution images were taken using HR-TEM, HAADF-STEM mode using JEOL, JEM-ARM 200F at 200 kV. Fourier-transform infrared (FT-IR) spectra were recorded on a Nicolet 6700 FT-IR spectrometer. Raman studies were performed using a Horiba HR 800 Raman system equipped with a 488 nm wavelength laser source. ^1^H NMR spectrum in DMSO-d_6_ solvent recorded at 500 MHz in a JEOL ECA-500. UV-Vis absorption spectra were recorded by a UV-Visible spectrophotometer (Lambda 25, Perkin-Elmer). GC-MS analysis were done using a Perkin Elmer GC Clarus 580 and MS Clarus SQ8S system. Photoluminescence measurements were carried out on a BioTek Synergy H4 Hybrid Microplate Reader. X-ray powder diffraction (XRD) patterns were measured by PANalytical Instruments D/max-3C using Cu-Kα radiation. Elemental analysis was done on Thermofinigann Flash 1112, each data was analysed for three times. X-Ray photoelectron spectroscopy (XPS) analysis was performed using a K-Alpha X-ray Photoelectron Spectrometer system (Surface Analysis, Thermo Scientific) with Al-Kα as a source. Zeiss LSM 700 confocal microscope was used for cellular morphological analysis. The images were collected at 405 nm diode laser as the excitation source.

**Cell culturing technique:**

SHSY5Y cells (human neuroblastoma cell line) were cultured in Eagle's Minimum Essential Medium (EMEM) and F12 medium supplemented with 1% Penicillin, 1% streptomycin, 1% non-essential amino acids, 10% heat inactivated FBS kept in 95% air and 5% CO_2_ atmosphere in a humidified incubator at 37°C.

**Cytotoxicity Assay:**

SHSY5Y cells were seeded in a 24-well plate with a cell density of 1×10^4^ cells/plate and suspended in EMEM for 24 hrs. Later, the medium was replaced containing different concentrations of CNPs, CNCs and CDs (0-80 mg/ml). Then plates were placed at 37°C in a humidified 5% CO_2_ and 95% air incubator. After 24 hrs the assay was performed to determine the cell viability using a micro plate reader.

**Fluorescence imaging by confocal microscopy:**

After the growth of SHSY5Y cells in the EMEM/F12 medium on 13 mm glass coverslips positioned inside a 12-well plate with a 1×10^4^ cells/coverslip, the media was replaced by another batch of media containing CNPs, CNCs and CDs (50 mg/ml). Such a mixture was incubated at 37°C for 4 hrs. After 4 hrs, the adherent cells were washed three times for the removal of excess nanoparticles. Fluorescence imaging was then done on a confocal microscope (Zeiss LSM 700) using a diode laser at 405 nm as the excitation source. Plan-Apochromat 20×/0.8 M27 as well as 63×/1.40 Oil M27 objectives were used.

**Calculation of sp^2^ cluster size from Raman spectrum:**

Tuinstra and Koenig relation demonstrates inverse linear dependence between La (sp^2^ cluster size) and I_D_/I_G_ ratio in carbon structures^1^. This further explains the disorderness in the carbon structures. The relation is:


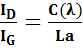
 **----------------------1a**

Where
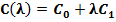
 **-------1b**

(C0=-12.6 nm), λ = 488nm, C1 =0.033

C (λ) = C0+ λC_1_ = 3.504

La = 3.504/0.936 = **3.74 nm ---------CDs**

La = 3.504/0.8315 = **4.214 nm---------CNCs**

La = 3.504/0.436 = **8.036 nm---------CNPs**

**Quantum yields measurements**

Quantum yields measurements was measured using 0.1% tryptophan in water (0.1 M phosphate buffer at pH 7.2) (Φ_F_ = 0.14) for CDs, CNPs and CNCs as a fluorescence standard. The absorbance of all 3 samples were measured at an emission wavelength range of 300-380 nm and matched with the standard^2^.

1. Lakowicz, J. R. Principles of Fluorescence Spectroscopy, 2nd Ed., 1999, Kluwer Academic/Plenum Publishers, New York.

The formula used to calculate quantum yield is as follows:


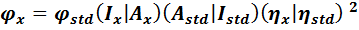
**-----------2**

Where
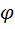
 is quantum yield,
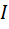
is the measured integrated emission intensity,
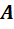
 is the optical density,
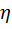
 is the refractive index and finally
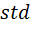
 refers to standard fluorescence reference of 0.1% tryptophan.


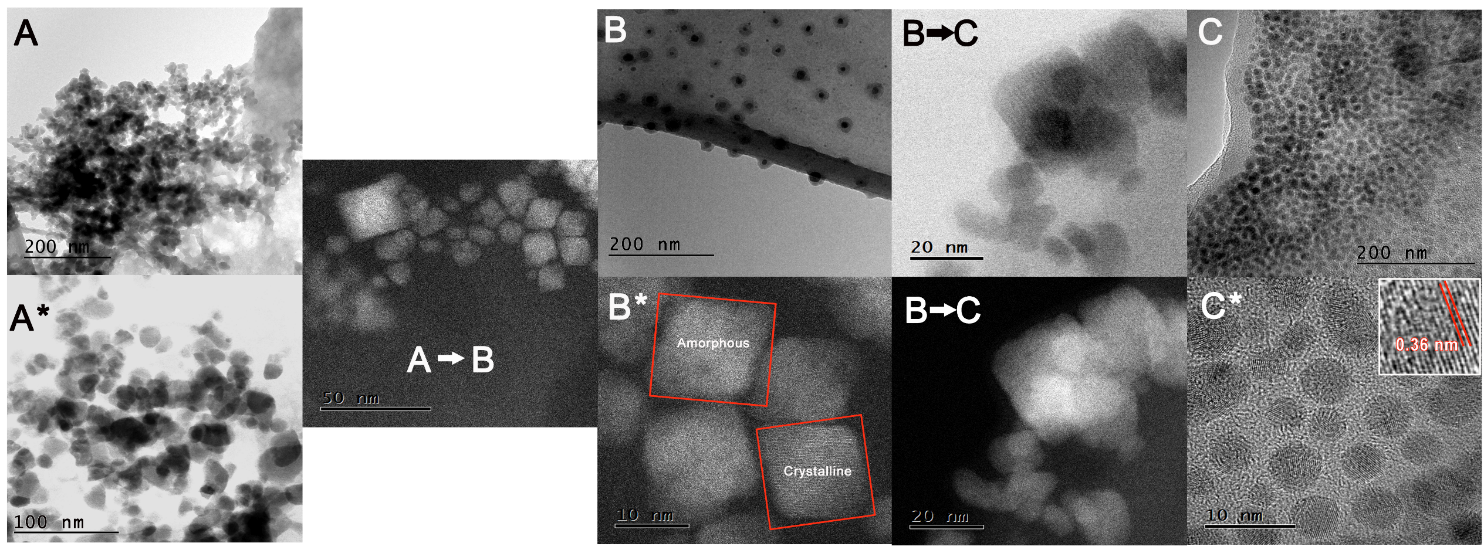


**Supplementary Fig S1.** A) TEM image of carbon nanoparticles (CNPs) interconnected to form a network, A*) HR-TEM image of CNPs, A to B) HAADF-STEM of intermediate transformation from CNPs to CNCs. B) TEM image of Carbon nanocubes (CNCs) encapsulated by graphitic shell, B*) HAADF-STEM of nanocuboidal seeds on which graphitic shells are formed, both amorphous and crystalline nanostructures are marked with red coloured square brackets, B to C) HAADF-STEM of transformation from CNCs to CDs, C) HR-TEM image of CDs, C*) HAADF-STEM image of CDs, after water-ethanol-chloroform centrifugation and dialysis, (inset shows IFFT of carbon dots exhibiting a lattice fringe width of 0.36 nm).


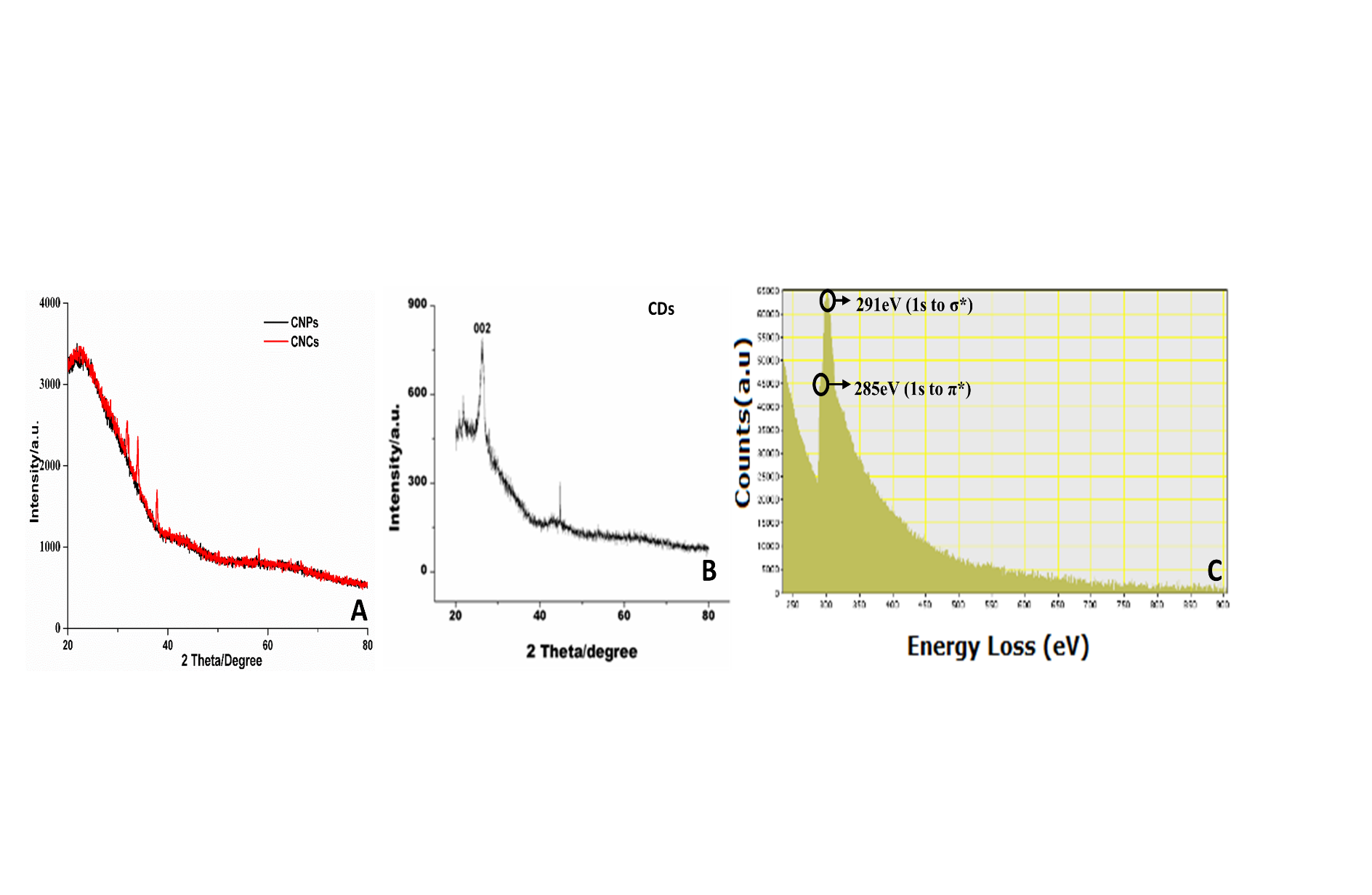


**Supplementary Fig S2.** XRD of CNPs, CNCs that corresponds to the graphitic structures and of CDs that corresponds to bulk graphite.


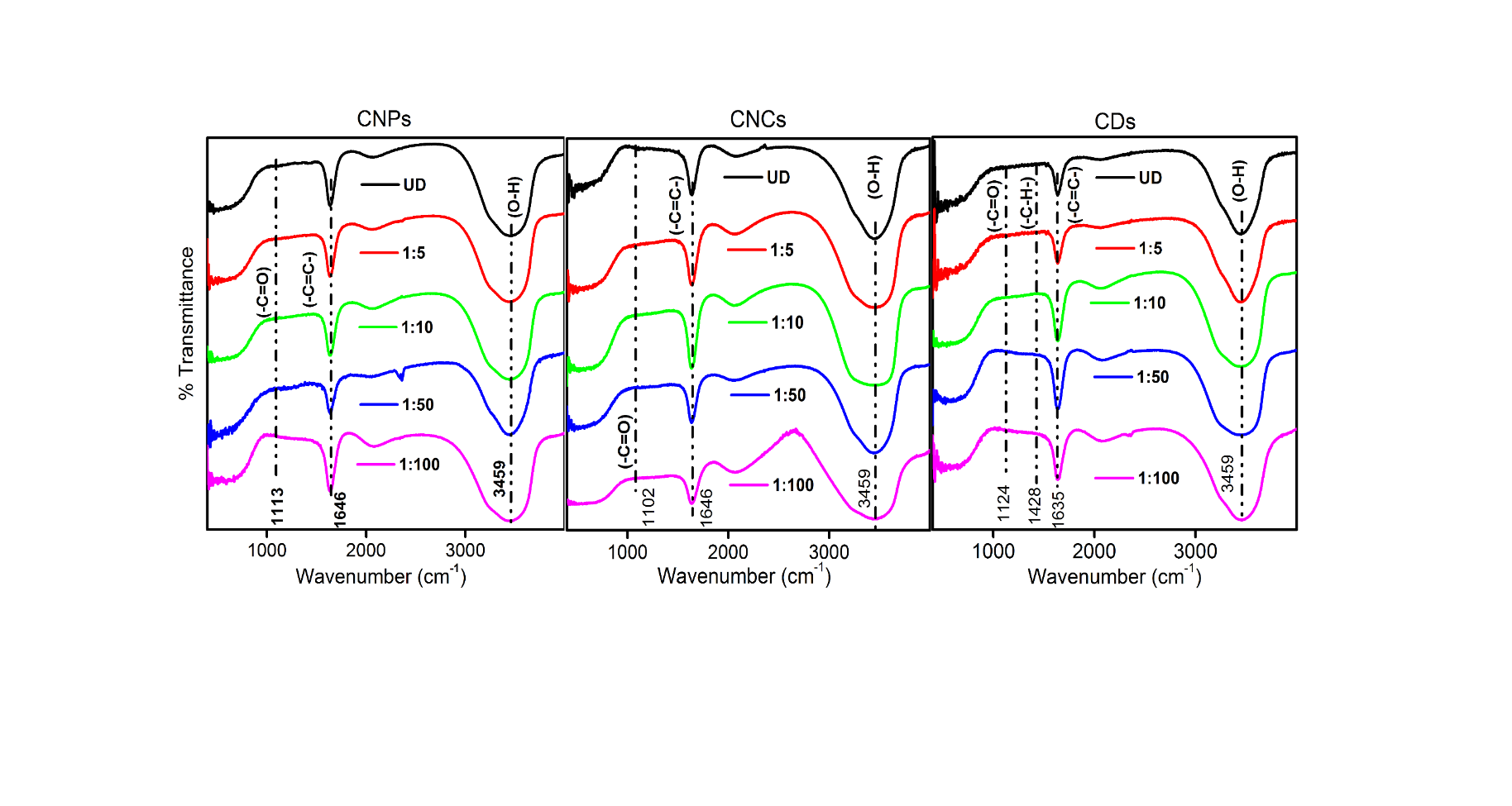


**Supplementary Fig S3.** FTIR spectra of undiluted (UD) and various dilutions from 1:5 to 1:100 of CNPs, CNCs and CDs

**
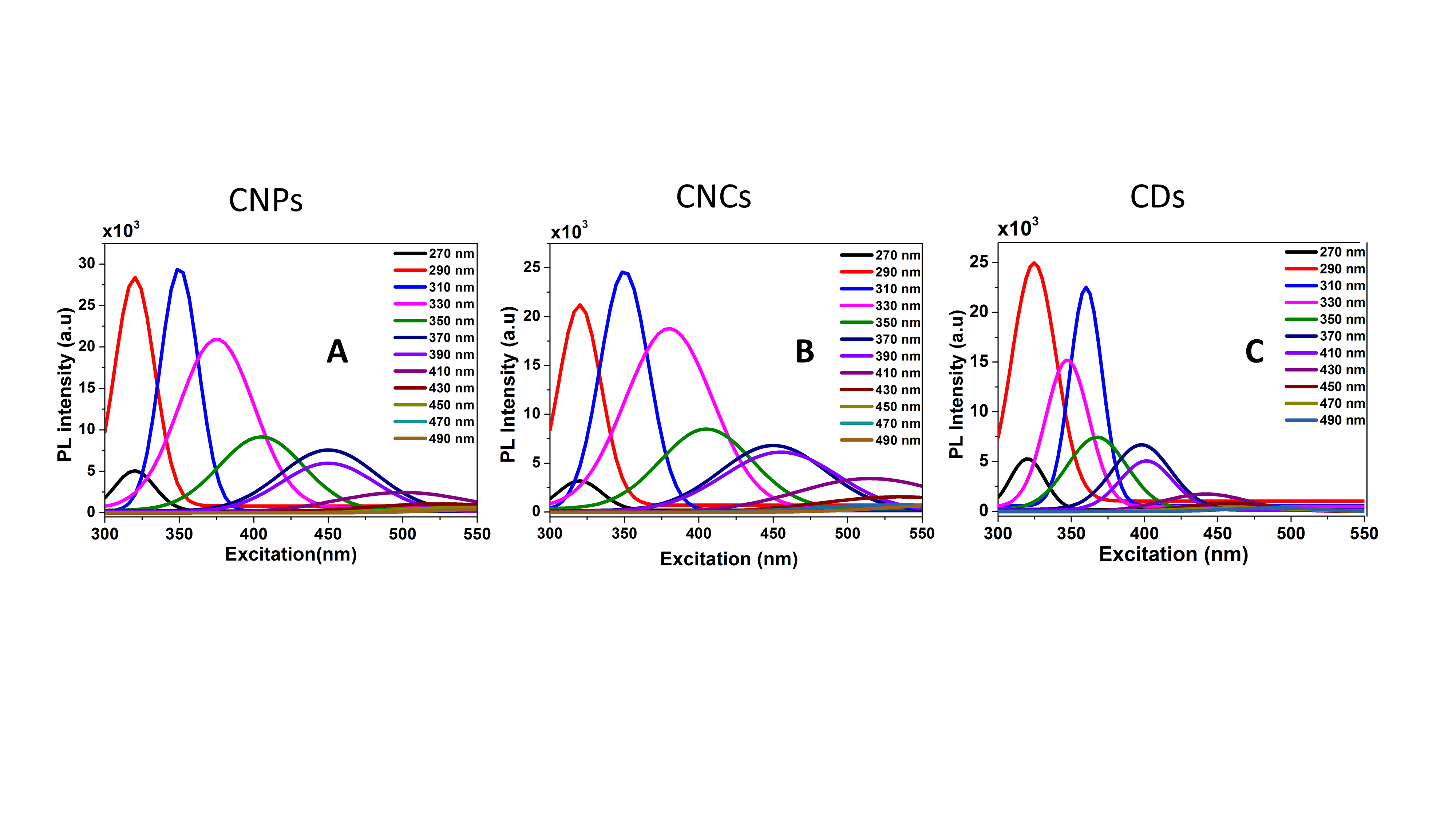
**


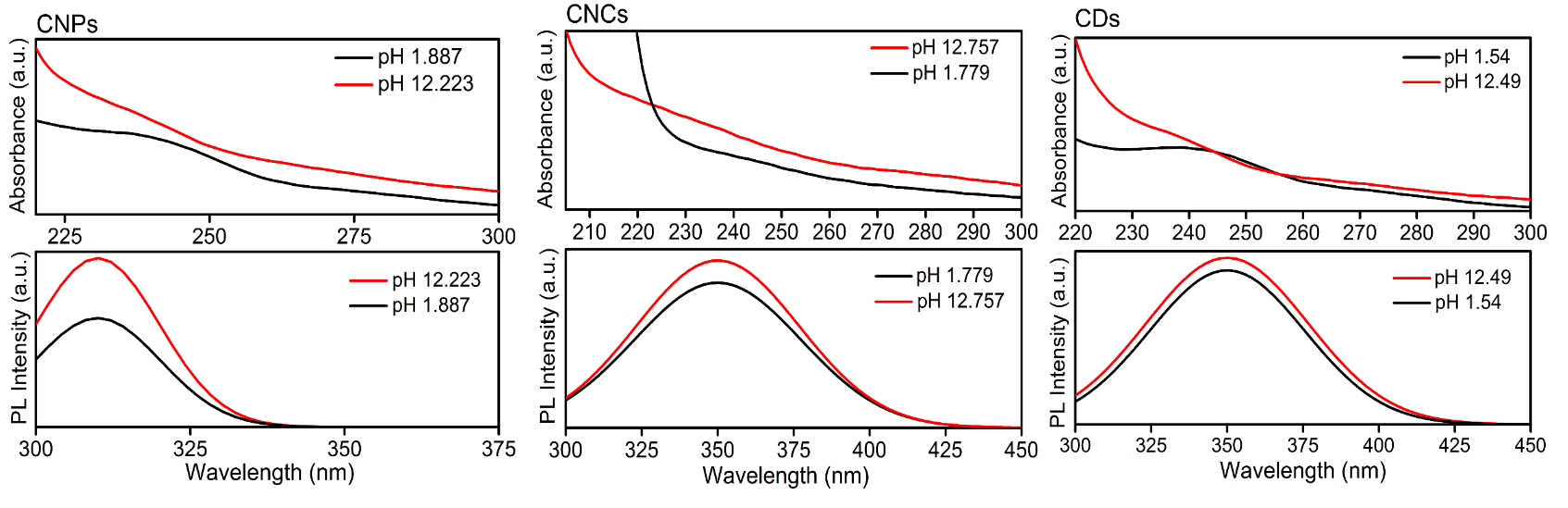
**Supplementary Fig S4.** 2D PL spectrum of A) CNPs, B) CNCs and C) CDs

**Supplementary Fig S5.** UV-Visible absorption spectra and photoluminescence spectrum of CNPs, CNCs and CDs from pH~1 to ~12.

**
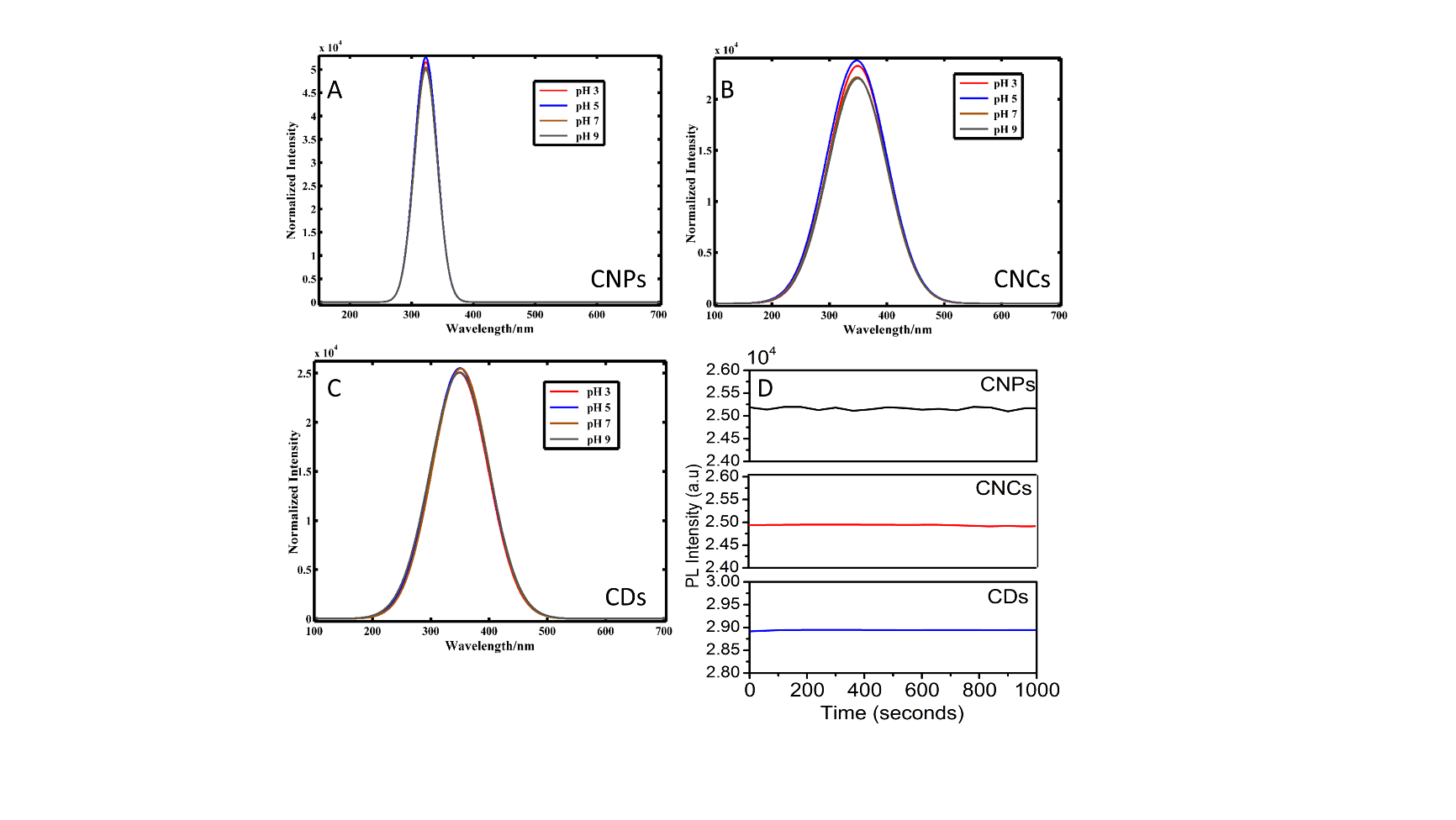
**

**Supplementary Fig S6**. A) Fluorescence emission spectra at 290 nm excitation wavelength at different pH 3, 5, 7 and 10 for CNPs, B) & C) fluorescence emission spectra at 310 nm excitation wavelength at different pH 3, 5, 7 and 10 for CNCs and CDs respectively. D) Photoemission stability of CNPs at 290 nm excitation wavelength for 1000 s, Photoemission stability of CNCs and CDs, at 310 nm excitation wavelength for 1000 s.

**
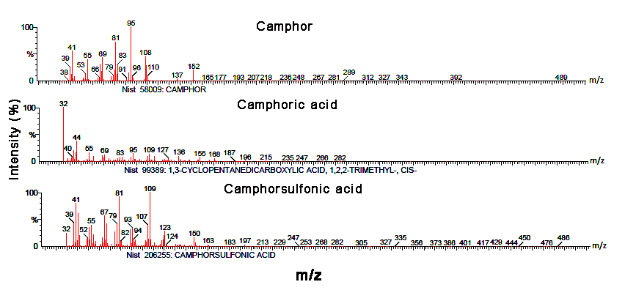
**

**Supplementary Fig S7.** GC-MS spectrum of precursor camphor, intermediate formed during the reaction such as camphoric acid and camphor sulfonic acid. The spectra was matched with NIST mass spectral database.

**
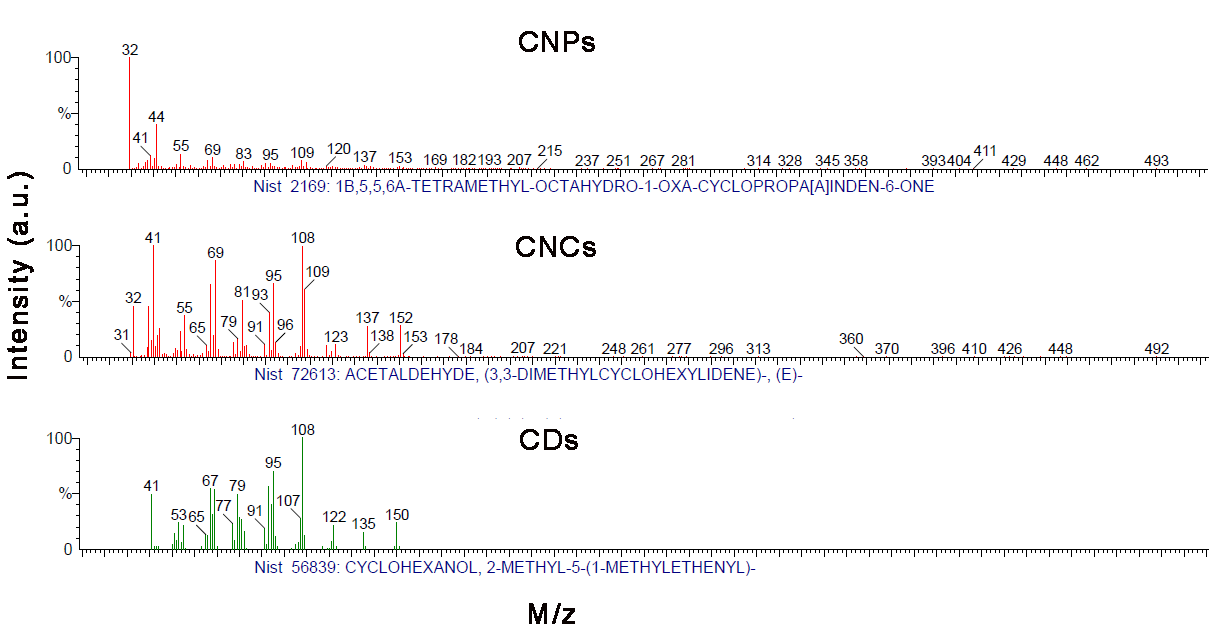
**

**Supplementary Fig S8.** GC-MS spectrum of CNPs, CNCs and CDs and the spectra was matched with NIST mass spectral database.

**
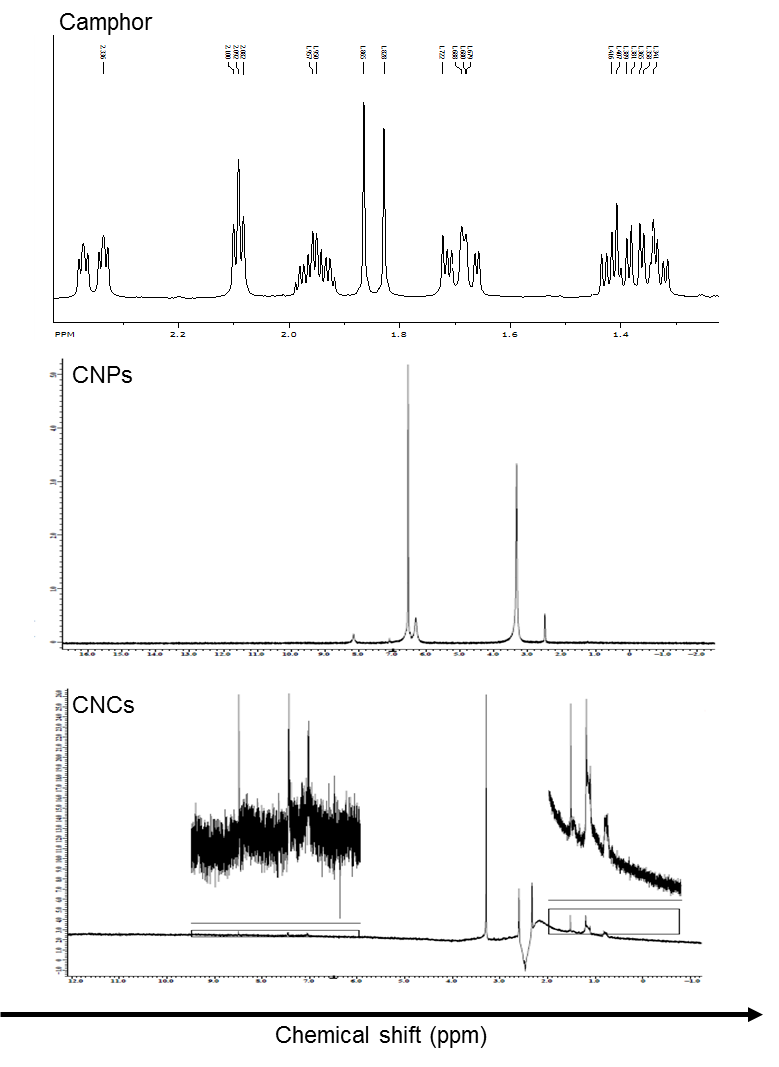
**

**Supplementary Fig S9.** ^1^H-NMR spectra of Camphor, CNCs and CNPs using DMSO-D_6_ as solvent.

**Supplementary Table S1**. Elemental analysis of Carbon and Hydrogen in Camphor, CNPs, CNCs and CDs.

| **Samples** | **C (wt%)** | **H (wt%)** |
| --- | --- | --- |
| **Camphor** | 78.90 | 10.59 |
| **CNPs** | 80.76 | 3.72 |
| **CNCs** | 83.36 | 5.49 |
| **CDs** | 86.12 | 7.76 |

**References**

1. Tuinstra, F. & Koenig, L. Raman Spectrum of Graphite. *J. Chem. Phys.* **53,** 1126–1130 (1970).

2. Lakowicz, J. R. *Principles of Fluorescence Spectroscopy Principles of Fluorescence Spectroscopy*. *Princ. Fluoresc. Spectrosc.* (2006). doi:10.1007/978-0-387-46312-4
